# Interfacial Free Energy Controlling Glass-Forming Ability of Cu-Zr Alloys

**DOI:** 10.1038/srep05167

**Published:** 2014-06-04

**Authors:** Dong-Hee Kang, Hao Zhang, Hanbyeol Yoo, Hyun Hwi Lee, Sooheyong Lee, Geun Woo Lee, Hongbo Lou, Xiaodong Wang, Qingping Cao, Dongxian Zhang, Jianzhong Jiang

**Affiliations:** 1 Division of Physical Metrology, Korea Research Institute of Standards and Science, Daejeon 305-340, Republic of Korea; 2 International Center for New-Structured Materials (ICNSM), and State Key Laboratory of Silicon Materials, Zhejiang University and Laboratory of New-Structured Materials, Department of Materials Science and Engineering, Zhejiang University, Hangzhou 310027, People's Republic of China; 3 Department of Chemical and Materials Engineering, University of Alberta, Edmonton, Alberta, T6G 2V4, Canada; 4 Pohang Accelerator Laboratory, Pohang 790-784, Republic of Korea; 5 Department of Science of Measurement, University of Science and Technology, Daejeon 305-333, Republic of Korea; 6 State Key Laboratory of Modern Optical Instrumentation, Zhejiang University, Hangzhou 310027, People's Republic of China; 7 These authors contributed equally to this work.

## Abstract

Glass is a freezing phase of a deeply supercooled liquid. Despite its simple definition, the origin of glass forming ability (GFA) is still ambiguous, even for binary Cu-Zr alloys. Here, we directly study the stability of the supercooled Cu-Zr liquids where we find that Cu_64_Zr_36_ at a supercooled temperature shows deeper undercoolability and longer persistence than other neighbouring compositions with an equivalent driving Gibbs free energy. This observation implies that the GFA of the Cu-Zr alloys is significantly affected by crystal-liquid interfacial free energy. In particular, the crystal-liquid interfacial free energy of Cu_64_Zr_36_ in our measurement was higher than that of other neighbouring liquids and, coincidently a molecular dynamics simulation reveals a larger glass-glass interfacial energy value at this composition, which reflects more distinct configuration difference between liquid and crystal phase. The present results demonstrate that the higher crystal-liquid interfacial free energy is a prerequisite of good GFA of the Cu-Zr alloys.

Understanding glass-forming ability (GFA)^1^,^2^,^3^ of metallic glasses (MGs) is a long-standing fundamental issue for basic science and their applications. With progress in strategies for alloying design, over 30 mm in diameter bulk metallic glasses (BMGs) prepared by directly copper mold cast technique were achieved in four alloy systems, i.e., 73 mm ZrCuAgAlBe^4^, 50 mm TiZrNiCuBe^5^, 40 mm PdNiCuP^6^, 35 mm LaCuAgAlCoNi^7^. However, the exact origin for the GFA in BMGs has not yet been completely understood^8^,^9^,^10^,^11^,^12^,^13^,^14^,^15^,^16^,^17^. Rather than the complexity in multi-component BMGs, binary Cu-Zr BMGs^11^ (its critical diameter is larger than 1 mm) have been intensively studied to figure out the GFA, because of its simplicity, and showing very sensitive dependence of composition on the critical size forming glassy phase, even narrow down to 1 at. %.

Though enormous studies^11^,^12^,^13^,^14^,^15^,^16^,^17^, clear explanations have not been suggested for the origin of the good GFA for the Cu-Zr BMGs. For instance, in thermodynamic point of view, Kwon et al.^16^ reported that the good GFA of Cu_64_Zr_36_ was attributed to the relatively small difference of Gibbs free energy between amorphous and crystal phases. Wang et al.^11^ pointed out that the GFA of Cu-Zr MGs was significantly influenced by the crystallization of competing phases. In addition, it was reported that the liquid structure of the Cu-Zr MGs could affect their thermal stability and the GFA; Yang et al.^15^ addressed that a relatively high atomic-packing efficiency of short range order (SRO) on the Cu_64_Zr_36_ BMG was attributed to the good GFA. On the other hand, in the kinetic point of view, Li et al.^12^ demonstrated that the composition forming the BMGs on Cu-Zr alloys had higher density than other Cu-Zr compositions. This suggests denser metallic glasses have higher viscosity in the supercooled state, resulting in the good GFA. Moreover, Bendert et al.^17^ suggested that the GFA of Cu-Zr alloys resulted from the different fragility at the supercooled liquid state and also near glass-transition temperature (T*_g_*), based on the measurement of thermal expansion coefficients. In other words, the stronger viscous behaviour of Cu-Zr liquid gives the better GFA. Nevertheless, the direct observation of such strong viscosity behaviour of the supercooled liquids forming Cu-Zr BMGs has not been reported yet. Therefore, the GFA is still ambiguous even for binary Cu-Zr MGs, although significant recent efforts have been devoted to study the GFA.

One of determinant factors to facilitate the good GFA that has not been considered in detail is the stability of supercooled Cu-Zr liquids. Since glasses are a freezing phase of deeply supercooled liquid, the better stability should give the deeper undercooling, resulting in the vitrification of the supercooled liquid. Therefore, we should directly study the stability of the supercooled Cu-Zr liquids. We report crystal-liquid interfacial free energy and glass-glass interfacial energy of Cu-Zr alloys, resulting in the stability of the supercooled Cu-Zr liquids using a containerless technique, i.e., electrostatic levitation (ESL), providing the deep supercooling, and using molecular dynamics (MD) simulation study. It is found that for a given composition the higher GFA, the higher undercoolability and the longer persistence of a supercooled liquid state. We provide compelling evidence to demonstrate that the interfacial free energy is a decisive factor to stabilize the supercooled liquid and thus affect the GFA, which is a novel approach to understand the GFA of the Cu-Zr alloys.

## Results

### Undercoolability and persistence of supercooled liquid

**Figure 1:**
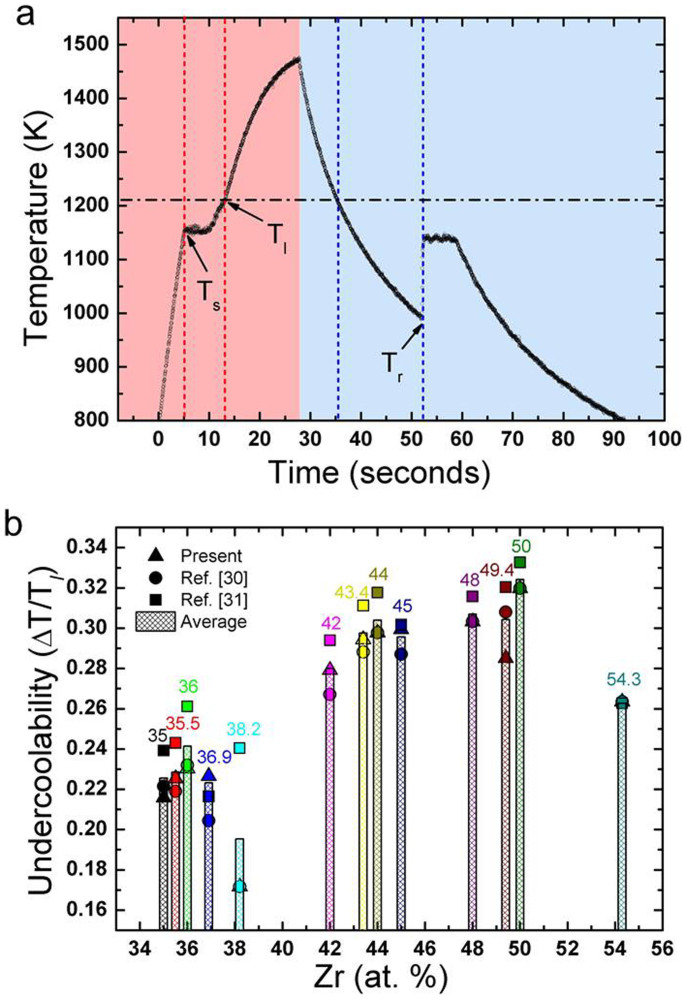
Time-Temperature curve of Cu_64_Zr_36_ and undercoolability of Cu_100-*x*_Zr*_x_* (*x* = 35–54.3 at.%) alloys. (a) A typical Time-Temperature curve of the Cu_64_Zr_36_ binary alloy by the electrostatic levitation (ESL) experiment, where *T_s_* is the solidus temperature, T*_l_* is the liquidus temperature, and T*_r_* is the recalescence temperature. (b) Undercoolability, ΔT/T*_l_* = (T*_l_* − T*_r_*)/T*_l_*, of Cu_100-*x*_Zr*_x_* (*x* = 35–54.3 at. %) as a function of the Zr concentration. Three different liquidus temperatures of Cu-Zr alloys are used to determine the undercoolability, i.e., T*_l_* from present ESL experiments (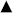), and from ref. 30 (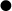) and ref. 31 (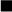) phase diagrams. Meshed column is the result of the average liquidus temperature from the three liquidus T*_l_*.

Figure 1a shows a representative heating and cooling curve of a Cu-Zr sample. The sample is heated over their liquidus temperature (T*_l_*) and cools down radiatively by turning off the heating lasers. Undercoolability is one of the signatures for the liquid stability, described by the degree of supercooling of the liquids, i.e., ΔT/T*_l_* = (T*_l_* − T*_r_*)/T*_l_*, where T*_l_* and T*_r_* are the liquidus and recalescence temperatures, respectively. Figure 1b shows the reduced undercooling values (ΔT/T*_l_*) of various Cu_100-*x*_Zr*_x_* (*x* = 35–54.3 at. %) alloy liquids. Interestingly, maxima are observed at Cu_64_Zr_36_ (ΔT/T*_l_* = 0.24 ± 0.01), Cu_56_Zr_44_ (ΔT/T*_l_* = 0.30 ± 0.01) and Cu_50_Zr_50_ (ΔT/T*_l_* = 0.32 ± 0.01), regardless different liquidus temperatures. The maxima in undercooling are consistent with the critical thickness or the GFA on Cu-Zr MGs^12^, and thermal expansion coefficient maxima in Cu-Zr liquids^17^. The maxima in undercooling mean that the supercooled liquids of Cu_64_Zr_36_, Cu_56_Zr_44_ and Cu_50_Zr_50_ have better stability, compared with neighbouring compositions. Based on the classical nucleation theory (CNT)^18^, the deeper undercooling reflects the higher nucleation barrier. Therefore, the liquids showing undercooling maxima (i.e., better stability) should be expected to show longer persistence at a same degree of the supercooled temperature than the neighbouring composition liquids.

**Figure 2:**
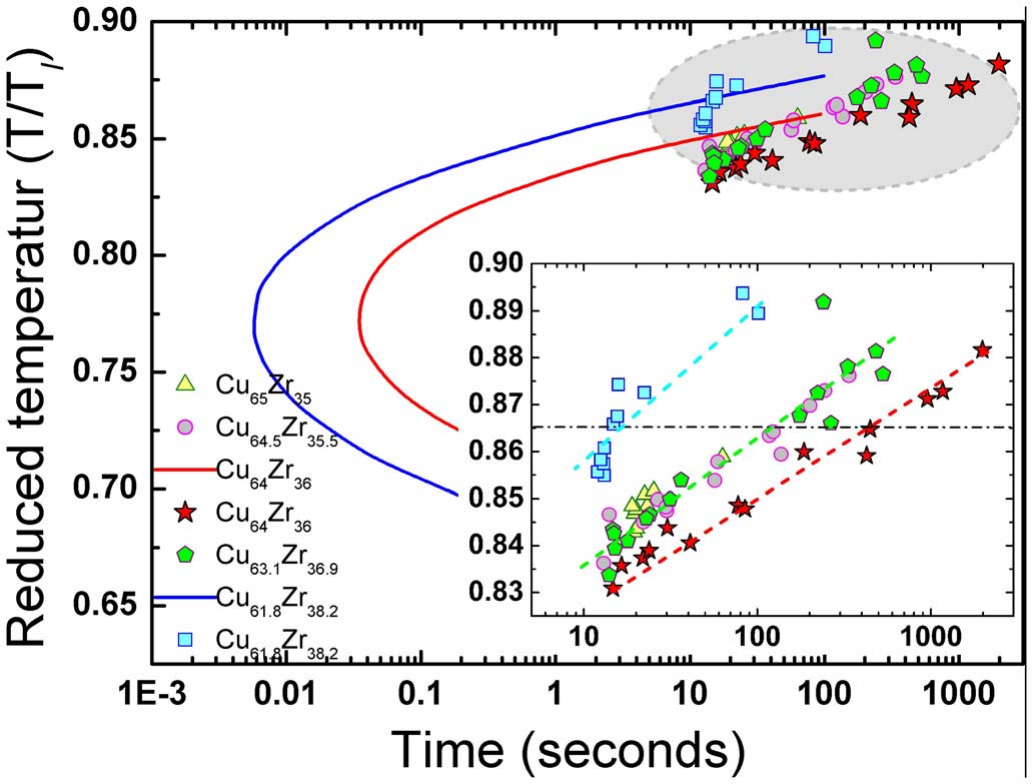
Reduced Time-Temperature-Transformation (TTT) diagram of Cu_100-*x*_Zr*_x_* (*x* = 35–38.2 at.%) alloys. T*_l_* at reduced temperature (T/T*_l_*) is the average value of the three different liquidus temperatures (T*_l_*: present, T*_l_*: ref. 30, T*_l_*: ref. 31).The blue and red lines are of calculated TTT curves from ref.^20^, and dots are of experimental results in present study using the ESL technique. The inset is magnified from the dots with dashed guide lines for eyes.

To verify the stability of the supercooled liquid as a function of time, we carried out Time-Temperature-Transformation (TTT) experiments. In particular, we focus on the Cu_64_Zr_36_, since the origin of the higher GFA for the most studied composition is still unclear. Figure 2 shows the TTT-diagram of Cu_100-*x*_Zr*_x_* (*x* = 35–38.2 at. %) liquids. At the same reduced supercooling temperature, *T*/*T_l_* = 0.865, as an example, Cu_64_Zr_36_ liquid indeed persists for much longer time 455 s in the supercooled liquid state than other neighbouring compositions, 124 s and 16 s for Cu_63.1_Zr_36.9_ and Cu_61.8_Zr_38.2_, respectively. This experimental observation is consistent with the calculated TTT-diagram of Cu_64_Zr_36_ (red) and Cu_61.8_Zr_38.2_ (blue) from ref. 19, although the exact values are different. According to the CNT, the nucleation barrier (ΔG*) resisting the crystallization is determined by two factors, crystal-liquid interfacial free energy (*σ*) and Gibbs free energy difference of liquid and crystal (Δ*G_v_*), i.e., ΔG* = 16*πσ*^3^/3(Δ*G_v_*)^2^ 18. Here, the same reduced temperature is assumed to give the same driving force for the nucleation (in case of Turnbull's approximation, Δ*G_v_* = Δ*H_f_*(1 − *T*/*T_l_*), where Δ*H_f_* is the fusion enthalpy). This assumption is reasonable because Δ*H_f_* should not be significantly different for the Cu-Zr liquids within such a small composition range (35–38.2 at. % of Zr). Accordingly, the longer persistence of the supercooled liquid at the same *T*/*T_l_* indicates that the stability of the supercooled liquids is decisively determined by the interfacial free energy (*σ*).

### Interfacial free energy

**Figure 3:**
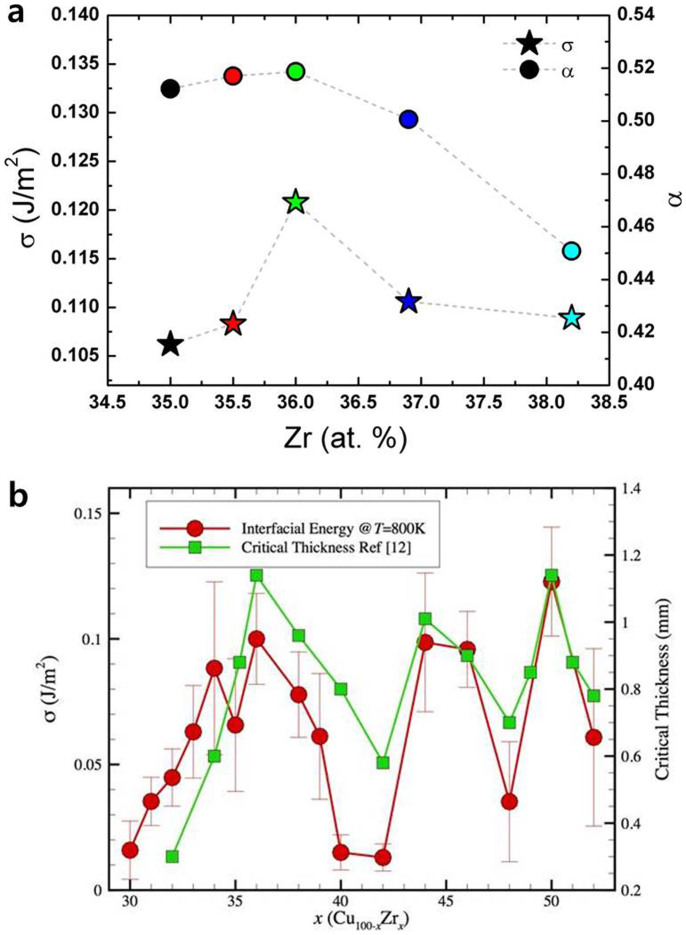
Interfacial free energies of Cu_100-*x*_Zr*_x_* (*x* = 35–38.2 at.%) alloys. (a) Estimated crystal-liquid interfacial free energies (*σ*) from the undercooling data in the ESL experiment using the classical nucleation theory (CNT) and Turnbull's coefficients (*α*) of Cu_100-*x*_Zr*_x_* (*x* = 35–38.2 at. %) alloys. (b) Calculated glass-glass interfacial energy of Cu-Zr metallic glasses (MGs) at 800 K as a function of Zr composition together with the critical thickness for the glass formation in the Cu-Zr alloy system reported in ref. 12.

Since the deeper undercooling and the longer persistence of the supercooled liquid Cu_64_Zr_36_ imply higher interfacial free energy, to confirm it, we estimate the crystal-liquid interfacial free energy from the undercooling data using the CNT. To initiate crystallization reaction, at least one critical nucleus should be formed in the supercooled liquid. This requires that the number of the critical nucleus in the supercooled liquid should be greater than one with a given liquid volume *V*, a steady state nucleation rate *I^s^*, and time *t* at a certain temperature *T*^18^, i.e., 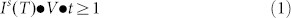
Here, the steady state nucleation rate *I^s^* per unit volume at temperature *T* is given by 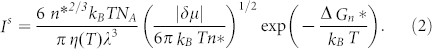
where *η*, *λ*, n*, *δμ*, *k_B_*, *N_A_* and ΔG_n_* are the viscosity, the average atomic jump distance, the number of atoms in the critical nucleus, the Gibbs free energy difference between the initial and final phases per atom, the Boltzmann's constant, the Avogadro number, and the work (i.e., nucleation barrier) of critical cluster formation, respectively. The work ΔG_n_* is determined by the Gibbs free energy difference Δ*G_v_* = *δμ/v*, the interfacial free energy (*σ*), and the average atomic volume (*v*). As expected, it is found that the Cu_64_Zr_36_ alloy, having the higher GFA^12^, exhibits the highest crystal-liquid interfacial free energy value in Figure 3, which is consistent with the undercoolability and TTT-diagram results in Figures 1b and 2. It should be mentioned that the density and viscosity of Cu-Zr liquids measured in this study show weak composition dependence, which is reasonable with the narrow composition range. This is consistent with a previous report^17^ that show no volume anomaly of Cu-Zr alloy liquids with compositions. Thus no composition effect of density and viscosity within the temperature range was found to estimate the interfacial free energy of Cu-Zr alloys.

In addition, the glass-glass interfacial energies for Cu_100-*x*_Zr*_x_* (*x* = 30–52 at. %) MGs were calculated at 800 K by molecular dynamics (MD) simulations in Figure 3b. It should be stressed that a prerequisite for calculating crystal-liquid interfacial free energies is the crystal structure, and the orientation of the crystal at the interface. Unfortunately, such information is not available as we change the composition of the alloys. And thus instead of directly calculating crystal-liquid interfacial free energies, we calculated the glass-glass interfacial energies of Cu-Zr MGs (see the details in Supporting Online Material). Since the difference of regularity or atomic packing density of short range order in bulk and surface of the glass is dependent of compositions, the interfacial energy between the glasses would show the dependence of composition. This gives the relation of short range order and GFA with composition. Although these data are not exactly the same as those for the crystal-liquid interfacial free energies, they still interestingly provide the same trends as the interfacial free energies of crystal-supercooled liquid of Cu_100-*x*_Zr*_x_* (*x* = 35–38.2 at. %) estimated from the undercooling data.

### Configurational differences causing interfacial free energy

Following the Turnbull's explanation of the stability of the supercooled liquid (or the high nucleation barrier) by crystal-liquid interface structure^20^, the higher interfacial free energy for Cu_64_Zr_36_ alloy implies the larger difference of the configurational ordering between the supercooled liquid and the crystal. Unlike crystal-gas interface, atoms in the liquid near a crystal show ordering, called neg-entropic ordering. When the atomic configurational ordering at the interface is more different from that of the crystal, the larger interfacial free energy forms, and thus leads to the high nucleation barrier^21^,^22^ as follows. 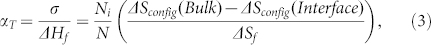
where *N_i_* is the number of atoms in the interface, *N* is the number of atoms in the crystal plane, Δ*S_config_*(*bulk*) is the configurational entropy of the bulk crystal, Δ*S_config_*(*interface*) is the configurational entropy of the interface, and Δ*S_f_* is the fusion entropy per atom. The higher Turnbull's coefficient (*α*) on Cu_64_Zr_36_ (*α_T_* = 0.519), as shown in Figure 3a, indicates that the configurational ordering between the crystal and the supercooled liquid of Cu_64_Zr_36_ differs from that of neighbouring compositions.

To further confirm the higher interfacial free energy for Cu_64_Zr_36_ alloy, we further carried out synchrotron X-ray diffraction measurements for Cu_100-*x*_Zr*_x_* (*x* = 35–38.2 at. %) MGs at ambient temperature. It is indeed found that the Cu_64_Zr_36_ MG had the highest dense-packing of icosahedral short range ordering (ISRO) than other compositions, which is consistent with the recently reported results for Cu-Zr MGs^15^. Similar to other studies^23^, a high fraction of icosahedral-like clusters in Cu_64_Zr_36_ MG was also detected after Voronoi tessellation analyses of the results obtained by reverse Monte Carlo (RMC) simulation to the experimental data^15^. Since the icosahedral short range order (ISRO) clearly differs from the short range orders in Cu-Zr crystal phases, the highest atomic packing on Cu_64_Zr_36_ MG could result in the large difference of configurational entropy between crystal and amorphous alloy. It is worth to note an opposite example which validates the conclusion; a liquid forming Ti-Zr-Ni icosahedral quasicrystal phase (*i*-phase) showed a lower interfacial free energy (*σ* = 0.06 J/m^2^) and *α_T_* = 0.32 with small undercooling (ΔT/T*_l_* = 0.1), since the both local orders of the liquid and *i*-phase are same local ordering, i.e, icosahedral order^24^,^25^.

The highest interfacial free energy on the Cu_64_Zr_36_ liquid and glass links with the higher atomic-packing efficiency on Cu_64_Zr_36_ BMG^15^, i.e., more difference of configurational ordering between the liquid and the crystal. Hence, the good GFA on Cu_64_Zr_36_ comparing with neighbouring compositions is attributed to the stability of supercooled liquid with the high interfacial free energy. Moreover, a recent simulation study^26^ revealed the importance of crystal-liquid interface structure for the GFA; the poorer crystal-liquid interfacial structure, the larger difference of local ordering between liquid and crystal, leading to the better GFA for Cu_50_Zr_50_ as compared with Ni_50_Al_50_^26^, which is consistent with this study.

## Discussion

We carried out molecular dynamics (MD) simulation to calculate glass-glass interfacial energies for Cu_100-*x*_Zr*_x_* (*x* = 30–52 at. %) MGs at 800 K (Fig.3b). Interestingly, the glass-glass interfacial energies at 800 K provide the same trends with the crystal-supercooled liquid interfacial free energy of Cu_100-*x*_Zr*_x_* (*x* = 35–38.2 at. %) estimated from undercooling data (Fig.3). Since the glass-glass interface is formed by welding two glasses, the interfacial energy reflects the different degree of randomness in short range order in bulk and interface of the glasses due to the more excessive volume on surface than in bulk of the glass. In other words, the surface has more defective short range ordering^27^. Therefore, when the glass has the better regularity or the higher packing density of the ISRO in bulk, the larger difference of the excessive volume between the bulk and the surface is given, which causes the higher interfacial energy. Accordingly, the higher interfacial energy of Cu_64_Zr_36_ compared with the neighbour compositions supports that the better regularity or higher packing density of ISRO results in the better GFA^15^. Moreover, other glass forming compositions, i.e., Cu_56_Zr_44_, and Cu_50_Zr_50_ also show higher glass-glass interfacial free energies.

One would expect that the glass-glass interfacial energy would eventually diminish at high temperatures. One might also expect that such temperature is sensitive to alloy compositions, i.e., a higher interfacial energy usually corresponds to a higher diminishing temperature because the reduction of the interfacial energy at the high temperature is closely related to the glass relaxation time and the higher interfacial energy may be related to a higher viscosity. However, further simulation needs to be carried out to confirm this hypothesis.

The C-curve in the TTT diagram is usually observed in bulk metallic glasses, due to the result of the competition of two characteristic times, i.e., *τ_n_* and *τ_D_* for nucleation and viscosity (or diffusion), respectively. Below a nose temperature in TTT diagram, *τ_D_* for viscosity property governs the GFA. In contrast, *τ_n_* for nucleation governs the GFA above the nose temperature. Although electrostatic levitation can easily achieve deeper supercooling state than other methods, the cooling rate by radiation on Cu-Zr alloys is not fast enough to bypass the nose temperature in TTT diagram. This prevents from verifying the reported characteristic viscosity behaviour (i.e., fragility)^17^ or the role of *τ_D_* to explain the good GFA on Cu-Zr alloys. Instead, we can clearly see the characteristics of *τ_n_* for nucleation with the higher undercoolability, the longer persistence of supercooled liquid state, and the higher interfacial free energy (i.e., nucleation barrier), which reflects the stability of the supercooled Cu-Zr liquids in figures 1, 2 and 3. Therefore, if a moderate cooling rate is given, the stability of supercooled Cu-Zr liquids should be kept, and the liquids can become glasses.

In conclusion, we investigate the origin of the GFA on a Cu-Zr alloy system. We found that Cu_64_Zr_36_ showed better undercoolability and stayed in supercooled liquid state for longer time, compared with neighbouring compositions. Under the same driving force, the longer persistence of the supercooled liquid on Cu_64_Zr_36_ revealed that interfacial free energy was the key factor for the stability of the supercooled liquid. The highest interfacial free energy on Cu_64_Zr_36_ estimated from both undercooling experiment and simulation supports this scenario, on comparing with the neighbouring compositions. Our findings could have an implication that the GFA for various compositions in a given alloy system is controlled by the interfacial free energy between crystal and supercooled alloy liquids. The higher interfacial free energy, the higher the GFA. This should certainly be validated in other MG systems.

## Methods

### Sample preparation

Spherical Cu_100-*x*_Zr*_x_* (*x* = 35–54.3 at. %) alloys were prepared by an arc-melting with high-purity copper (Cu, 99.99%) and zirconium (Zr, 99.995%) slugs under high-purity argon (Ar, 99.9999%) atmosphere. To avoid reaction with oxygen, a piece of Zr was used as an oxygen getter. The samples were melted at least four times for the homogeneity of each composition.

### Undercooling experiment and data acquisition

The levitated samples with 2–3 mm in diameter were melted under high vacuum condition in the range of 10^−6^–10^−7^ Torr by CO_2_ heating lasers with triangle arrangement for symmetrical heating. Detailed description of the electrostatic levitation (ESL) equipment is given in refs.^28^ and 29. The samples were heated over their liquidus temperature (T*_l_*) and cooled radiatively by turning off the heating lasers. The sample temperature was measured for every 6 ms by two infrared pyrometers with InGaAs detector operating at 1.55 μm wavelength (547–1973 K) and 1.6 μm wavelength (773–2773 K). The spectral emissivity (E*_λ_*) of Cu-Zr alloy is determined with the known solidus temperature (T*_s_*) by the phase diagram of the binary Cu-Zr alloys^30^.

### Synchrotron X-ray scattering experiment

For the structural study, the synchrotron X-ray scattering experiments were carried out in Hasylab in Germany^15^ and in PAL in Republic of Korea. Related thermophysical parameters and MD simulations details are given in Supporting Online Material.

## Author Contributions

G.W.L., D.-H.K. and J.Z.J. designed the project. D.-H.K., H.B.Y. and H.H.L. performed and supported the experimental work. G.W.L., S.H.L. and D.-H.K. analysed the experimental data. H.Z., H.B.L. and X.D.W. performed simulations. H.Z., H.B.L., X.D.W., Q.P.C., D.X.Z. and J.Z.J. analysed the simulation data. G.W.L., D.-H.K. and J.Z.J. wrote the paper.

## Supplementary Material

Supplementary Information Supporting Online Material for Interfacial Free Energy Controlling Glass-Forming Ability of Cu-Zr Alloys
