# Supplementary material for: Interfacial Free Energy Controlling Glass-Forming Ability of Cu-Zr Alloys
Source: Sci Rep. 2014 Jun 4;4:5167. doi: 10.1038/srep05167 (PMC4044622; doi:10.1038/srep05167)
Supplement: Supplementary Information — Supporting Online Material for Interfacial Free Energy Controlling Glass-Forming Ability of Cu-Zr Alloys [file srep05167-s1.pdf]

Supporting Online Material for

Interfacial Free Energy Controlling Glass-Forming Ability of Cu-Zr Alloys

Dong-Hee Kang[1], Hao Zhang[2,3#], Hanbyeol Yoo[1], Hyun Hwi Lee[4], Sooheyong Lee[1], Geun Woo Lee[1,5*], Hongbo Lou[2], Xiaodong Wang[2], Qingping Cao[2], Dongxian Zhang[6] and Jianzhong Jiang[2*]

[1]*Division of Physical Metrology, Korea Research Institute of Standards and Science, Daejeon 305-340, Republic of Korea*

[2]*International Center for New-Structured Materials (ICNSM), and State Key Laboratory of Silicon Materials, Zhejiang University and Laboratory of New-Structured Materials, Department of Materials Science and Engineering, Zhejiang University, Hangzhou 310027, People's Republic of China*

[3]*Department of Chemical and Materials Engineering, University of Alberta, Edmonton, Alberta, T6G 2V4, Canada*

[4]*Pohang Accelerator Laboratory, Pohang 790-784, Republic of Korea*

[5]*Department of Science of Measurement, University of Science and Technology, Daejon, 305-333, Republic of Korea*

[6]*State Key Laboratory of Modern Optical Instrumentation, Zhejiang University, Hangzhou, 310027, People's Republic of China*

[*]e-mail: gwlee@kriss.re.kr and jiangjz@zju.edu.cn

[#]contributes equally with the first author.

1. Thermophysical parameters to estimate the interfacial free energy.

Table SI shows the interfacial free energy and Turnbull coefficient, and related thermophysical parameters used in this study. Density of supercooled liquids was measured by image analysis during cooling in ESL. The boundary of the sample image was fitted by 6th order Lengendre polynomial which was a well-known method [1, 2]. Viscosity measurements were carried out using the resonant oscillation drop method [3, 4]. The resonant oscillation of the samples at different temperatures was induced by applying the ac electric field, and the decay time-constant of the oscillation was obtained by the fitting of decay function. Specific heat can be obtained by Stephan-Boltzmann (SB) law, $mC_p(T)\frac{dT}{dt} = -\sigma_B A\varepsilon_T(T^4 - T_o{}^4)$, since the liquids cool down radiatively under high vacuum in the ESL. From the SB law, we can get an accurate ratio of C_p/ε_T. If total hemispherical emissivity (ε_T) is given, the C_p can be calculated. However, the ε_T has not been reported for Cu-Zr liquids so far. Therefore, in this study, we used spectral emissivity which is used to measure sample temperature. Then, fusion enthalpy (ΔH_f) was calculated by multiplying C_p and temperature rising ΔT from the recalescence temperature T_r on nucleating to the plateau temperature T_s in Figure 1 in the main text. Although this cannot give accurate values, this consistent calculation for all compositions is enough to show the trend in interfacial free energy with compositions. Moreover, the TTT study clearly showing the longest persistence of the supercooled liquid on $Cu_{64}Zr_{36}$ means that ΔH_f does not significantly affect the interfacial free energy among the narrow composition range (35 – 38.2 at. % of Zr).

Table SI. Interfacial free energies (**σ**), Turnbull's coefficients (**α_{LS}**), and critical radius of nucleus (**r^***) for the $Cu_{100-x}Zr_x$ (x = 35 – 38.2 at.%) alloys, and thermophysical parameters used.

	**σ**	**α_{LS}**	**r^***	**ρ (T_l)**	**C_p**	**ΔH_f**	**log η =A + B/(T-C)**	**emissivity**
	(J/m^2)		(nm)	(g/cm^3)	(J/mol·K)	(kJ/mol)	(Pa·s)	
**$Cu_{65}Zr_{35}$**	0.106± 0.001	0.512± 0.001	1.4198	7.3	62.2	8.148	A = -3.1964<br>B = 646.03<br>C = 730	0.36
**$Cu_{64.5}Zr_{35.5}$**	0.108± 0.001	0.517± 0.001	1.4017	7.3	60.6	8.241	A = -2.9241<br>B = 553.24<br>C = 728	0.36
**$Cu_{64}Zr_{36}$**	0.121± 0.001	0.519± 0.001	1.3133	7.2	60.1	9.255	A = -2.9493<br>B = 573.75<br>C = 726	0.36

**$Cu_{63.1}Zr_{36.9}$**	0.111± 0.001	0.501± 0.001	1.3809	7.2	62.0	8.804	A = -3.0714<br>B = 633.58<br>C = 722	0.35
**$Cu_{61.8}Zr_{38.2}$**	0.109± 0.001	0.451± 0.001	1.3745	7.2	62.3	9.656	A = -3.007<br>B = 606.09<br>C = 717	0.38

2. **Calculations of glass-glass interfacial energies of Cu-Zr amorphous alloys** (Figs. S1-S4)

The glass-glass interfacial energies of various Cu-Zr amorphous alloys (AAs) were estimated using molecular dynamics simulations. The atomic interactions for Cu-Zr were described using the Mendelev [5] form of an Embedded Atom Method (EAM) [6] potential. The MD simulations utilized LAMMPS [7], which was developed at the Sandia National Laboratories. In order to generate atomic configurations of $Cu_{100-x}Zr_x$ (x = 30 – 52 at.%) amorphous alloys for molecular dynamics simulations, we started with a perfect copper single crystal containing 8,000 atoms with a box size of about 4.3×8.6×4.3nm, and then randomly substituted certain percentage (x %) of Cu atoms by Zr atoms. The initial model was held at the temperature of 2000 K (a temperature that is well above the melting point of the alloy) for 3 ns (time step was 1 fs) for relaxation, in which the simulation time was long enough to ensure the system to reach a homogeneous liquid. The model was then performed a rapid quench to T = 50K at a cooling rate of 160 K/ns. The above simulations were performed at zero pressure with the NPT ensemble using periodic boundary conditions. This method combined the Parrinello-Rahman algorithm [8] for controlling the pressure and box size, and the Nose-Hoover method [9, 10] for maintaining constant temperature. MD simulations continued to run for additional 1 ns at T = 50 K to obtain equilibrium thermodynamics properties such as volume and potential energy. In order to calculate the interfacial energy of the amorphous alloys, we first cut the bulk amorphous alloys along the center plane of the model perpendicular to y-axis and then separated the two parts from each other by a distance of 2 nm to ensure the atoms on the surfaces do not interact with each other. The new simulation box was then thermally relaxed at 1000 K for 2 ns with the Canonical ensemble (NVT) using periodic boundary

conditions. This thermal relaxation was to ensure the atomic configurations on the free surfaces to reach equilibrium. After relaxation, system was quickly quenched to T = 50 K at a cooling rate of 250 K/ns. MD simulation continues to run for additional 1 ns at T = 50 K to obtain equilibrium potential energy of the system with free surfaces, then we move the two free surfaces together to form an interface at T = 50 K, followed by a relaxation of 6 ns with NPT ensemble (only control the box size along y-axis to ensure a zero pressure) using periodic boundary conditions. The interfacial energy is then defined as $s = \left(E_{\text{Interfacial}} - E_{\text{Bulk}}\right) / A$, where $E_{\text{Interfacial}}$ is the total potential energy of the system with one interface, E_{Bulk} is the total potential energy of the system before cutting and A the cross-section area in perpendicular to y-axis. To calculate the interfacial energy of amorphous alloys at high temperatures, we quickly raise the temperature of the system to 800 K from 50 K and then relax the system isothermally at 800 K for 12 ns under hydrostatic pressure of 3 GPa. We performed three runs for each composition for a better statistics. Since large internal stress might build up within system during solidification under large supercooling, it is not unreasonable to apply 3 GPa pressure in current calculation. From self-diffusivity as a function of external hydrostatic pressure, it is confirmed that the external hydrostatic pressure 3 GPa will not fundamentally change the self-diffusion behavior of atoms in those alloys. Similar technique has been used in the study of the thermal stability of interfacial energy [11].

Figure S1 illustrates an equilibrium atomic configuration of a selected composition $Cu_{64}Zr_{36}$ amorphous alloy at T = 50 K, where blue and yellow spheres indicate Cu and Zr atoms, respectively. Figure S2 shows the potential energies of Cu and Zr in a selected $Cu_{64}Zr_{36}$ amorphous alloy as a function of the position relative to interface at T = 50 K. The potential energy of each component was normalized by its potential energy in bulk amorphous alloys. It is clear that an increase of the potential energy in the interface will result in an excess interfacial energy, mainly due to the structural change in the interface region. Figure S3 shows interfacial energy of Cu-Zr amorphous alloys as a function of Zr

composition at T =50 K. The interfacial energy has a range of 0.34 – 0.56 J/m^2 for the studied $Cu_{100-x}Zr_x$ (x = 30 – 52 at. %) amorphous alloys at 50 K. It is found that the interfacial energy non-monotonically changes with composition. Three local maxima (about x = 36, 46 and 50 at. %) and two minima (about x = 42 and 48 at. %) are clearly detected within the uncertainty. By comparing these data with the critical thickness for glass formation in the Cu-Zr alloy system reported in Ref. 12, the composition dependence of the interfacial energy matches well with the composition dependence of the critical thickness for glass formation. We further calculate the interfacial energy of Cu-Zr amorphous alloys at 800 K in Fig. S4. The interfacial energy decreases as temperature increases. However, the variation of interfacial energy with composition is still persistent even at high temperature. The values at 800 K deduced here are very similar to the interfacial free energies of crystal-supercooled liquid of $Cu_{100-x}Zr_x$ (x = 35 – 38.5 at. %) estimated from undercooling data.

This finding mentioned above is further confirmed in a second Zr-Cu-Al alloy system, which was selected due to the fact that both reliable empirical potential [13] and experimental measurements for critical thickness [14] are available to this system. The trend, i.e., the higher the interfacial free energy of amorphous alloy, the higher GFA is also held for 6 studied Zr-Cu-Al alloys (0.314 J/m^2 at 50 K and 0.075 J/m^2 at 600 K for $Zr_{46}Cu_{44}Al_{10}$, 0.375 J/m^2 at 50 K and 0.054 J/m^2 at 600 K for $Zr_{49}Cu_{44}Al_7$, 0.465 J/m^2 at 50 K and 0.116 J/m^2 at 600 K for $Zr_{48}Cu_{45}Al_7$, 0.346 J/m^2 at 50 K and 0.080 J/m^2 at 600 K for $Zr_{49}Cu_{45}Al_6$, 0.383 J/m^2 at 50 K and 0.101 J/m^2 at 600 K for $Zr_{48}Cu_{46}Al_6$ and 0.254 J/m^2 at 50 K and 0.015 J/m^2 at 600 K for $Zr_{49}Cu_{46}Al_5$), further supporting that the interfacial energy of amorphous alloys indeed play an important role for the glass forming ability in alloy systems. We believe that interfacial energy of amorphous alloys should link with the variation of local atomic structures (e.g., type of polyhedron, connection of various polyhedra etc.) in amorphous alloys. More studies of atomic structures are still needed to address the origin of the composition dependence of

interfacial energy for studied amorphous alloys.

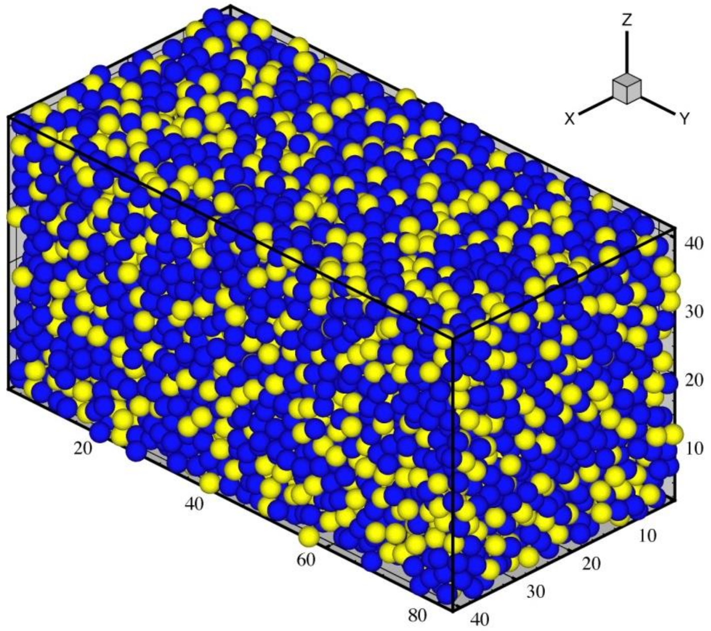

Figure S1 An equilibrium atomic configuration of a selected composition $Cu_{64}Zr_{36}$ amorphous alloy at T=50 K, in which blue and yellow spheres indicate Cu and Zr atoms, respectively.

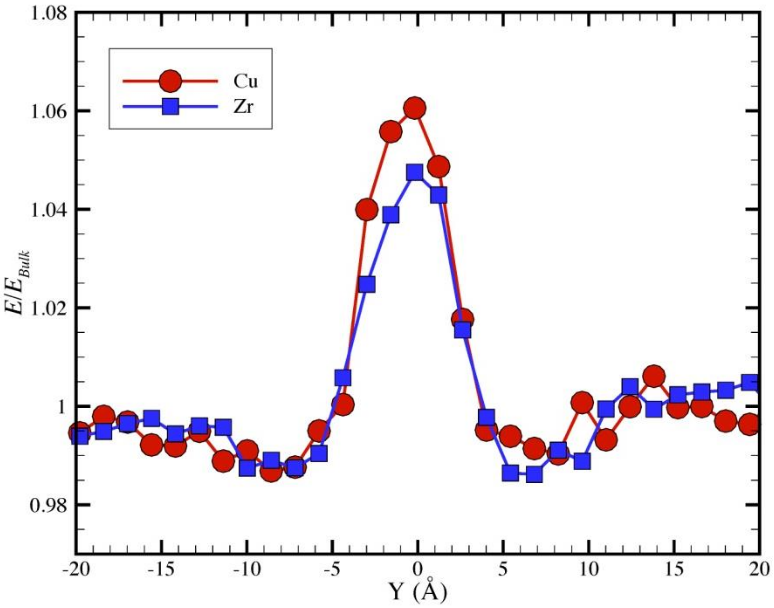

Figure S2 Potential energy of Cu and Zr in $Cu_{64}Zr_{36}$ amorphous alloy as a function of interface position at T = 50 K.

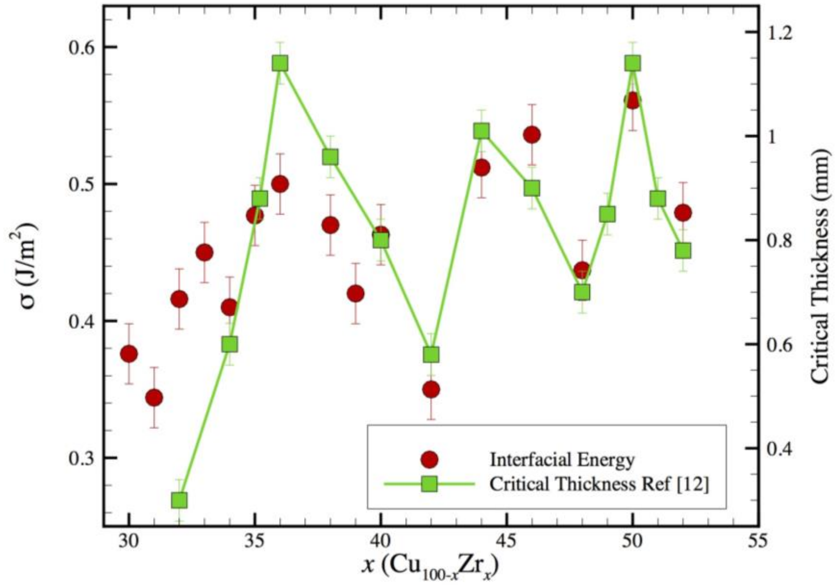

Figure S3 Interfacial energy of Cu-Zr amorphous alloys at 50 K as a function of Zr composition together with the critical thickness for glass formation in the Cu-Zr alloy system reported in Ref. 12.

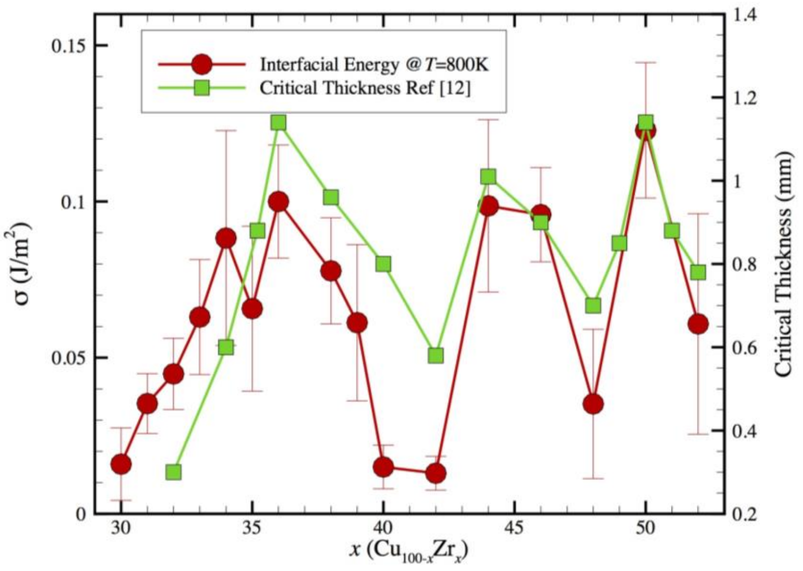

Figure S4 Interfacial energy of Cu-Zr amorphous alloys at 800 K as a function of Zr composition together with the critical thickness for glass formation in the Cu-Zr alloy system reported in Ref. 12.

References

1. Bradshaw, R. C., Schmidt, D. P., Rogers, J. R., Kelton, K. F. & Hyers, R. W. Machine vision for high-precision volume measurement applied to levitated containerless material processing. *Rev. Sci. Instru.* **76**, 125108 (2005).

2. Lee, G. W., Jeon, S., Park, C. & Kang, D.-H. Crystal-liquid interfacial free energy and thermophysical properties of pure liquid Ti using electrostatic levitation: Hypercooling limit, specific heat, total hemispherical emissivity, density, and interfacial free energy. *J. Chem. Thermodynamics* **63**, 1–6 (2013).

3. Hyers, R. W. *et al.* Surface tension and viscosity of quasicrystal-forming Ti-Zr-Ni alloys. *Int, J. Thermophys.* **25**, 1155–1162 (2004).

4. Ishikawa, T., Paradis P. -F. & Yoda, S. Noncontact surface tension and viscosity measurements of rhenium in the liquid and undercooled states. *Appl. Phys. Lett.* **85**, 5866–5868 (2004).

5. Mendelev, M. I. *et al.* Development of suitable interatomic potentials for simulation of liquid and amorphous Cu-Zralloys. *Philos. Mag.* **89**, 967–987 (2009).

6. Foiles, S. M., Baskes, M. I. & Daw, M. S. Embedded-atom-method functions for the fcc metals Cu, Ag, Au, Ni, Pd, Pt, and their alloys. *Phys. Rev. B* **33**, 7983–7991 (1986).

7. Plimpton, S. Fast Parallel Algorithms for short-range molecular dynamics. *J. Comput. Phys.* **117**, 1–19 (1995).

8. Parrinello, M. & Rahman, A. Polymorphic transitions in single crystals: A new molecular dynamics method. *J. Appl. Phys.* **52**, 7182–7190 (1981).

9. Nosé, S., A unified formulation of the constant temperature molecular dynamics methods. *J. Chem. Phys.* **81**, 511–519 (1984).

10. Hoover, W. G. Canonical dynamics: equilibrium phase-space distributions. *Phys. Rev. A* **31,** 1695–1697 (1985).

11. Ritter, Y., Şopu, D., Gleiter, H. & Albe, K. Structure, stability and mechanical properties of internal interfaces in $Cu_{64}Zr_{36}$ nanoglasses studied by MD simulations. *Acta Mater.* **59**, 6588–6593 (2011).

12. Li, Y., Guo, Q., Kalb, J. A. & Thompson, C. V. Matching glass-forming ability with the density of the amorphous phase. *Science* **322**, 1816–1819 (2008).

13. Cheng, Y. Q., Ma, E. & Sheng, H. W. Atomic level structure in multicomponent bulk

metallic glass. *Phys. Rev. Lett.* **102**, 245501 (2009).
14. Wang, D., Tan, H. & Li, Y. Multiple maxima of GFA in three adjacent eutectics in Zr-Cu-Al alloy system – A metallographic way to pinpoint the best glass forming alloys. *Acta Mater*. **53**, 2969–2979 (2005).
